# Supplementary material for: Does Forest Therapy Have Physio-Psychological Benefits? A Systematic Review and Meta-Analysis of Randomized Controlled Trials
Source: Int J Environ Res Public Health. 2022 Aug 24;19(17):10512. doi: 10.3390/ijerph191710512 (PMC9518146; doi:10.3390/ijerph191710512)
Supplement: Supplementary file 1 [file ijerph-19-10512-s001.zip › ijerph-1827956-supplementary.pdf]

Table S1. Summary of risk of bias assessment for randomized controlled trials (*n* = 17) [a]

First Author, year	Random sequence generation	Allocation concealment	Blinding of participants and personnel	Blinding of outcome assessment	Incomplete outcome data	Selective reporting	Other bias	Overall risk of bias [b]
Bang,2016 [10]	Low	Low	High	Low	Low	Low	Low	High
Brown,2014 [11]	Low	Low	Unclear	Low	Low	Low	Low	Unclear
Calogiuri,2016 [36]	High	Unclear	Unclear	Low	Low	Low	Low	High
Chun,2017 [21]	Low	Low	Unclear	Low	Low	Low	Low	Unclear
Grazuleviciene,2016 [37]	Unclear	Unclear	Unclear	Unclear	Low	Low	Low	Unclear
Huber,2019 [22]	Low	Low	Unclear	Low	Low	Low	Low	Unclear
Jia,2016 [15]	Low	Low	Unclear	Low	High	Low	Low	High
Lee,2011 [39]	Low	Low	Low	Low	Low	Low	Low	Low
Lee,2014 [40]	Low	Low	Low	Low	Low	Low	Low	Low
Mao,2017 [41]	Low	Low	Unclear	Low	High	Low	Low	High
Mao(a),2012 [14]	Low	Low	Unclear	Low	High	Low	Low	High
Mao(b),2012 [23]	Low	Low	Unclear	Low	High	Low	Low	High
Niedermeier,2017 [12]	Low	Low	Unclear	Low	Low	Low	Low	Unclear
Shin,2012 [19]	Low	Low	Unclear	Low	High	Low	Low	High
Song,2019 [20]	Low	Low	Unclear	Low	High	Low	Low	High
Wu,2020 [13]	Low	Low	Unclear	Low	Low	Low	Low	Unclear
Zeng,2020 [38]	Low	Low	Unclear	Low	High	Low	Low	High

[a] Assessed using the Cochrane Collaboration's Risk of Bias Tool; [b] Overall risk of bias is Low if all domains are rated as low, High if at least one domain is assessed as high, and Unclear if at least one domain is assessed as unclear and no domains are assessed as high.
